# Management training programs in healthcare: effectiveness factors, challenges and outcomes

**DOI:** 10.1186/s12913-024-11229-z

**Published:** 2024-08-07

**Authors:** Lucia Giovanelli, Federico Rotondo, Nicoletta Fadda

**Affiliations:** 1 Department of Economics and Business, University of Sassari (Italy), Via Muroni 25, Sassari, 07100 Italy; 2 Department of Humanities and Social Sciences, University of Sassari (Italy), Via Roma 151, 07100 Sassari, Italy

**Keywords:** Management training programs, Healthcare professionals, Factors of effectiveness, Challenges, Outcomes

## Abstract

**Background:**

Different professionals working in healthcare organizations (e.g., physicians, veterinarians, pharmacists, biologists, engineers, etc.) must be able to properly manage scarce resources to meet increasingly complex needs and demands. Due to the lack of specific courses in curricular university education, particularly in the field of medicine, management training programs have become an essential element in preparing health professionals to cope with global challenges. This study aims to examine factors influencing the effectiveness of management training programs and their outcomes in healthcare settings, at middle-management level, in general and by different groups of participants: physicians and non-physicians, participants with or without management positions.

**Methods:**

A survey was used for gathering information from a purposive sample of professionals in the healthcare field attending management training programs in Italy. Factor analysis, a set of ordinal logistic regressions and an unpaired two-sample t-test were used for data elaboration.

**Results:**

The findings show the importance of diversity of pedagogical approaches and tools and debate, and class homogeneity, as effectiveness factors. Lower competencies held before the training programs and problems of dialogue and discussion during the course are conducive to innovative practice introduction. Interpersonal and career outcomes are greater for those holding management positions.

**Conclusions:**

The study reveals four profiles of participants with different gaps and needs. Training programs should be tailored based on participants’ profiles, in terms of pedagogical approaches and tools, and preserve class homogeneity in terms of professional backgrounds and management levels to facilitate constructive dialogue and solution finding approach.

**Supplementary Information:**

The online version contains supplementary material available at 10.1186/s12913-024-11229-z.

## Background

Several healthcare systems worldwide have identified management training as a precondition for developing appropriate strategies to address global challenges such as, on one hand, poor health service outcomes in front of increased health expenditure, particularly for pharmaceuticals, personnel shortages and low productivity, and on the other hand in terms of unbalanced quality and equal access to healthcare across the population [1]. The sustainability of health systems itself seems to be associated with the presence of leaders, at all levels of health organizations, who are able to correctly manage scarce resources to meet increasingly complex health needs and demands, at the same time motivating health personnel under an increasing amount of stress and steering their behaviors towards the system’s goals, in order to drive the transition towards more decentralized, interorganizational and patient-centered care models [2].

Recently, professional training as an activity aimed at increasing learning of new capabilities (reskilling) and improving existing ones (upskilling) during the lifetime of individuals (lifelong learning) has been identified by the European Commission as one of the seven flagship programs to be developed in the National Recovery and Resilience Plans (NRRP) to support the achievement of European Union’s goals, such as green and digital transitions, innovation, economic and social inclusion and occupation [3]. As a consequence, many member states have implemented training programs to face current and future challenges in health, which often represents a core mission in their NRRPs.

The increased importance of developing management training programs is also related to the rigidity and focalization of university degree courses in medicine, which do not provide physicians with the basic tools for fulfilling managerial roles [4]. Furthermore, taking on these roles does not automatically mean filling existing gaps in management capabilities and skills [5]. Several studies have demonstrated that, in the health setting, management competencies are influenced by positions and management levels as well as by organization and system’s features [6, 7]. Hence, training programs aimed at increasing management competencies cannot be developed without considering these differences.

To date, few studies have focused on investigating management training programs in healthcare [8]. In particular, much more investigation is required on methods, contents, processes and challenges determining the effectiveness of training programs addressed to health managers by taking into account different environments, positions and management levels [1]. A gap also exists in the assessment of management training programs’ outcomes [9]. This study aims to examine factors influencing the effectiveness and outcomes of management training, at the middle-management level, in healthcare. It intends to answer the following research questions: which factors influence the management training process? Which relationships exist between management competencies held before the program, factors of effectiveness, critical issues encountered, and results achieved or prefigured at the end of the program? Are there differences, in terms of factors of effectiveness, challenges and outcomes, between the following groups of management training programs’ participants: physicians and non-physicians, participants with or without management positions?

### Management training in healthcare

Currently, there is a wide debate about the added value of management to health organizations [10] and thus about the importance of spreading management competencies within health organizations to improve their performance. Through a systematic review, Lega et al. [11] highlighted four approaches to examine the impact of management on healthcare performance, focusing on management practices, managers’ characteristics, engagement of professionals in performance management and organizational features and management styles.

Although findings have not always been univocal, several studies suggest a positive relationship between management competencies and practices and outcomes in healthcare organizations, both from a clinical and financial point of view [12]. Among others, Vainieri et al. [13] found, in the Italian setting, a positive association between top management’s competencies and organizational performance, assessed through a multidimensional perspective. This study also reveals the mediating effect of information sharing, in terms of strategy, results and organization structure, in the relationship between managerial competencies and performance.

The key role of management competencies clearly emerges for health executives, who have to turn system policies into a vision, and then articulate it into effective strategies and actions within their organizations to steer and engage professionals [14–19]. However, health systems are increasingly complex and continually changing across contexts and health service levels. This means the role of health executives is evolving as well and identifying the capacities they need to address current and emerging issues becomes more difficult. For instance, a literature review conducted by Figueroa et al. [20] sheds light on priorities and challenges for health leadership at three structural levels: macro context (international and national), meso context (organizations) and micro context (individual healthcare managers).

Doctor-managers are requested to carry both clinical tasks and tasks related to budgeting, goal setting and performance evaluation. As a consequence, a growing stream of research has speculated whether managers with a clinical background actually affect healthcare performance outcomes, but studies have produced inconclusive findings. In relation to this topic, Sarto and Veronesi [21] carried out a literature review showing a generally positive impact of clinical leadership on different types of outcome measures, with only a few studies reporting negative impacts on financial and social performance. Morandi et al. [22] focused on doctor-managers who have become middle managers and investigated the potential bias in performance appraisal due to the mismatch between self-reported and official performance data. At the individual level, the role played by managerial behavior, training, engagement, and perceived organizational support was analyzed. Among others indications they suggested that training programs should be revised to reduce bias in performance appraisal. Tasi et al. [23] conducted a cross-sectional analysis of the 115 largest U.S. hospitals, divided into physician-led and non-physician-led, which revealed that physician-led hospital systems have higher quality ratings across all specialities and more inpatient days per hospital bed than non-physician-led hospitals. No differences between the groups were found in total revenue and profit margins. The main implication of their study is that hospital systems may benefit from the presence of physician leadership to improve the quality and efficiency of care delivered to patients as long as education and training are able to adequately prepare them. The main issue, as also observed by others [4, 24], is that university education in medicine still includes little focus on aspects such as collaborative management, communication and coordination, and leadership skills. Such a circumstance motivates the call for further training. Regarding the implementation of training programs, Liang et al. [1] have recently shown how it is hindered, among others, by a lack of sufficient knowledge about needed competencies and existing gaps. Their analysis, which focuses on senior managers from three categories in Chinese hospitals, shows that before commencing the programs senior managers had not acquired adequate management competencies either through formal or informal training. It is worth noticing that significant differences exist between hospital categories and management levels. For this reason, they recommend using a systemic approach to design training programs, which considers different hospital types, management levels and positions. Yarbrough et al. [6] examined how competence training worked in healthcare organizations and the competencies needed for leaders at different points of their careers at various organizational levels. They carried out a cross-sectional survey of 492 US hospital executives, whose most significant result was that competence training is effective in healthcare organizations.

Walston and Khaliq [25], from a survey of 2,001 hospital CEOs across the US concluded that the greatest contribution of continuing education is to keep CEOs updated on technological and market changes that impact their current job responsibilities. Conversely, it does not seem to be valued for career or succession planning. About the methods of continuing education, an increasing use of some internet-based tools was found. Walston et al. [26] identified the factors affecting continuing education, finding, among others, that CEOs from for-profit and larger hospitals tend to take less continuing education, whereas senior managers' commitment to continuing education is influenced by region, gender, the CEO's personal continuing education hours and the focus on change.

Furthermore, the principles that inspire modern healthcare models, such as dehospitalization, horizontal coordination and patient-centeredness, imply the increased importance of middle managers, within single structures but also along clinical pathways and projects, to create and sustain high performances [27–29].

Whaley and Gillis [8] investigated the development of training programs aimed at increasing managerial competencies and leadership of middle managers, both from clinical and nonclinical backgrounds, in the US context. By adopting the top managers’ perspective, they found a widespread difficulty in aligning training needs and program contents. A 360° assessment of the competencies of Australian middle-level health service managers from two public hospitals was then conducted by Liang et al. [7] to identify managerial competence levels and training and development needs. The assessment found competence gaps and confirmed that managerial strengths and weaknesses varied across management groups from different organizations. In general, several studies have shown that leading at various organizational levels, in healthcare, does not necessarily require the same levels and types of competencies.

Liang et al. [30] explored the core competencies required for middle to senior-level managers in Victorian public hospitals. By adopting mixed methods, they confirmed six core competencies and provided guidance to the development of the competence-based educational approach for training the current and future management workforce. Liang et al. [31] then focused on the poorly investigated area of community health services, which are one of the main solutions to reducing the increasing demand for hospital care in general, and, in particular, in the reforms of the Australian health system. Their study advanced the understanding of the key competencies required by senior and mid-level managers for effective and efficient community health service delivery. A following cross-sectional study by AbuDagga et al. [32] highlighted that some community health services, such as home healthcare and hospice agencies, also need specific cultural competence training to be effective, in terms of reducing health disparities.

Using both qualitative and quantitative methods, Liang et al. [33] developed a management competence framework. Such a framework was then validated on a sample of 117 senior and middle managers working in two public hospitals and five community services in Victoria, Australia [34]. Fanelli et al. [35] used mixed methods to identify the following specific managerial competencies, which healthcare professionals perceive as crucial to improve their performance: quality evaluation based on outcomes, enhancement of professional competencies, programming based on process management, project cost assessment, informal communication style and participatory leadership.

Loh [5], through a qualitative analysis conducted in Australian hospitals, examined the motivation behind the choice of medically trained managers to undertake postgraduate management training. Interesting results stemming from the analysis include the fact that doctors often move into management positions without first undertaking training, but also that clinical experience alone does not lead to required management competencies. It is also interesting to remark that effective postgraduate management training for doctors requires a combination of theory and practice, and that doctors choose to undertake training mostly to gain credibility.

Ravaghi et al. [36] conducted a literature review to assess the evidence on the effectiveness of different types of training and educational programs delivered to hospital managers. The analysis identifies a set of aspects that are impacted by training programs. Training programs focus on technical, interpersonal and conceptual skills, and positive effects are mainly reported for technical skills. Numerous challenges are involved in designing and delivering training programs, including lack of time, difficulty in employing competencies in the workplace, also due to position instability, continuous changes in the health system environment, and lack of support by policymakers. One of the more common flaws concerns the fact that managers are mainly trained as individuals, but they work in teams. The implications of the study are that increased investments and large-scale planning are required to develop the knowledge and competencies of hospital managers. Another shortage concerns the outcome measurement of training programs, which is a usually neglected issue in the literature [9]. It also emerges that the training programs performing best are specific, structured and comprehensive.

Kakemam and Liang [2] conducted a literature review to shed light on the methods used to assess management competencies, and, thus, professional development needs in healthcare. Their analysis confirms that most studies focus on middle and senior managers and demonstrate great variability in methods and processes of assessment. As a consequence, they elaborate a framework to guide the design and implementation of management competence studies in different contexts and countries.

In the end, the literature has long pointed out that developing and strengthening the competencies and skills of health managers represent a core goal for increasing the efficiency and effectiveness of health systems, and management training is crucial for achieving such a goal [37]. The reasons can be summarized as follows: university education has scarcely been able to provide physicians and, in general, health operators, with adequate, or at least basic, managerial competencies and skills; over time, professionals have been involved in increasingly complex and rapidly changing working environments, requiring increased management responsibilities as well as new competencies and skills; in many settings, for instance in Italy, delays in the enforcement of law requiring the attendance of specific management training courses to take up a leadership position, hindered the acquisition of new competencies and the improvement of existing ones by those already managing health organizations, structures and services.

For the purposes of this study, management competencies refer to the possession and ability to use skills and tools for service organization and service planning, control and evaluation, evidence-informed decision-making and human resource management in the healthcare field.

### Management training in the Italian National Health System

The reform of the Italian National Health System (INHS), implemented by Legislative Decree No. 502/1992 and inspired by neo-managerial theories, introduced the role of the general manager and assigned new responsibilities to managers.

However, the inadequate performance achieved in the first years of the application of the reform highlighted the cultural gap that made the normative adoption of managerial approach and tools unproductive on the operational level. Legislation evolved accordingly, and in order to hold management positions, management training became mandatory. Decree-Law No. 583/1996 (converted into Law No. 4/1997) provided that the requirements and criteria for access to the top management level were to be determined. Therefore, Presidential Decree No. 484/1997 determined these requirements and also the requirements and criteria to access the middle-management level of INHS’ healthcare authorities. This regulation also imposed the acquisition of a specific management training certificate, dictated rules concerning the duration, contents, and teaching methods of management training courses issuing this certificate, and indicated the requirements for attendance. Immediately afterwards, Legislative Decree No. 229/1999 amended the discipline of medical management and health professions and promoted continuous training in healthcare. It also regulated management training, which became an essential requirement for the appointments of health directors and directors of complex structures in the healthcare authorities, for the categories of physicians, dentists, veterinarians, pharmacists, biologists, chemists, physicists and psychologists.

The second pillar of the INHS reform was the regionalization of the INHS. Therefore, the Regions had to organize the courses to achieve management training certificates on the basis of specific agreements with the State, which regulated the contents, the methodology, the duration and the procedures for obtaining certification. The State-Regions Conference approved the first interregional agreement on management training in July 2003, whereas the State-Regions Agreement of 16 May 2019 regulated the training courses. The mandatory contents of the management training outlined the skills and behaviors expected from general managers and other top management key players (Health Director, Administrative Director and Social and Health Director), but also for all middle managers.

## Methods

A survey was used to gather information from a purposive sample of professionals in the healthcare field taking part in management training programs. In particular, a structured questionnaire was submitted to 140 participants enrolled in two management programs organized by an Italian university: a second-level specializing master course and a training program carried out in collaboration with the Region. The programs awarded participants the title needed to be appointed as a director of a ward or administrative unit in a public healthcare organization, and share the same scientific committee, teaching staff, administrative staff and venue. The respondents’ profile is shown in Table 1.

**Table 1:** Respondents’ profile

	*N*	*%*
***Gender***		
Male	62	44.29
Female	78	55.71
***Age***		
20—29	1	0.71
30—39	22	15.71
40—49	59	42.14
50—59	40	28.57
> 60	18	12.86
***Educational background***		
Medicine	83	59.29
Health professions	9	6.43
Economics and management	12	8.57
Pharmacy	6	4.29
Law	7	5.00
Engineering	3	2.14
Veterinary medicine	19	13.57
Other	1	0.71
***Management position***		
No	82	58.57
Yes	58	41.43
***Field of work***		
Healthcare	135	96.43
Other	5	3.57

It is worth pointing out that the teaching staff is characterized by diversity: teachers have different educational and professional backgrounds, are practitioners or academics, and come from different Italian regions.

The questionnaire was submitted and completed in presence and online between November 2022 and February 2023. All participants decided to take part in the analysis spontaneously and gave their consent, being granted total anonymity.

The questionnaire, which was developed for this study and based on the literature, consisted of 64 questions shared in the following five sections: participant profile (10 items), management competencies held by participants before the training program (4 items), effectiveness factors of the training program (23 items), challenges to effectiveness (10 items), and outcomes of the training program (17 items) (an English language version of the questionnaire is attached to this paper as a supplementary file). In particular, the second section aimed to shed light on the participants’ situation regarding management competencies held before the start of the training program and how they were acquired; the third section aimed to collect participants’ opinions regarding how the program was conducted and the factors influencing its effectiveness; the fourth section aimed to collect participants’ opinions regarding the main obstacles encountered during the program; and the fifth section aimed to reveal the main outcomes of the program in terms of knowledge, skills, practices and career.

Except for those of the first section, which collected personal information, all the items of the next four categories – management competencies, effectiveness factors, challenges and outcome — were measured through a 5-point Likert scale. To ensure that the content of the questionnaire was appropriate, clear and relevant, a pre-testing was conducted in October 2022 by asking four academics and four practitioners, both physicians and not, with and without management positions, to fill it out. The aim was to understand whether the questionnaire really addressed the information needs behind the study and was easily and correctly understood by respondents. Therefore, the four individuals involved in the pre-testing were asked to fill it out simultaneously but independently, and at the end of the compilation, a focus group that included them and the three authors was used to collect their opinions and suggestions. After this phase, the following changes were made: in the ‘Participant profile’ section, ‘Veterinary medicine’ was added to the fields accounting for the ‘Educational background’ (item 3); in Sect. 2, it was decided to modify the explanation given to ‘basic management competencies’ and align it to what required by Presidential Decree No. 484/1997; in Sect. 3, item 25 was added to catch a missing aspect that respondents considered important, and brackets were added to the description of items 15, 16 and 29 to clarify the concepts of mixed and homogenous class and pedagogical approaches and tools; in Sect. 4, in the description of item 40, the words ‘find the energy required’ were added to avoid confusion with items 38 and 39, whereas brackets were added to items 41 and 45 to provide more explanation; in Sect. 5, brackets were added to the description of item 51 to increase clarity, and the last item was divided into two (now items 63 and 64) to distinguish the training program’s impact on career at different times.

With reference to the methods, first, a factor analysis based on the principal component method was conducted within each section of the questionnaire (except for the first again), in order to reduce the number of variables and shed light on the factors influencing the management training process. Bartlett's sphericity test and the Kaiser–Meyer–Olkin (KMO) value were performed to assess sampling adequacy, whereas factors were extracted following the Kaiser criterion, i.e., eigenvalues greater than unity, and total variance explained. The rotation method used was the Varimax method with Kaiser normalization, except for the second section (i.e., management competencies held by participants before the training program) that), which did not require rotation since a single factor emerged from the analysis. Bartlett's sphericity test was statistically significant (*p* < 0.001) in all sections, KMO values were all greater than 0.65 (average value 0.765), and the total variances explained were all greater than 65% (average value of approximately 70.89%), which are acceptable values for such analysis.

Second, a set of ordinal logistic regressions were performed to assess the relationships existing between management competencies held before the start of the course, effectiveness factors, challenges, and outcomes of the training program.

The factors that emerged from the factor analysis were used as independent variables, whereas some significant outcome items accounting for different performance aspects were selected as dependent variables: improved management competencies, innovation practices, professional relationships, and career prospects. Ordered logit regressions were used because the dependent variables (outcomes) were measured on ordinal scales. Some control variables for the respondent profiles were included in the regression models: age, gender, educational background, management position, and working in the healthcare field.

With the aim of understanding which explanatory variables could exert an influence, a backward elimination method was used, adopting a threshold level of significance values below 0.20 (*p* < 0.20). Table 4 shows the results of regressions with independent variables obtained following the criterion mentioned above. All four models respected the null hypothesis, which means that the proportional odds assumption behind the ordered logit regressions had not been rejected (*p* > 0.05). Third and last, an unpaired two-sample t-test was used to examine the differences between groups of participants in the management training programs selected based on two criteria: physicians and non-physicians, and participants with or without management positions.

## Results

First, descriptive statistics is useful for understanding the aspects participants considered the most and least important by category. This can be done by focusing on the items of the four sections of the questionnaire (except for the first one depicting participant profiles) that were given the highest and lowest scores at the sample level and by different groups of participants (physicians and non-physicians, participants with or without management positions). Table 2 summarizes the mean values and standard deviations by group of these higher and lower scores. Focusing on management competencies, all groups reported having mainly acquired them through professional experience, except for non-physicians who attributed major significance to postgraduate training programs, with a mean value of 3.05 out of 5. All groups agreed on the poor role of university education in providing management competencies, with mean values for the sample and all four groups below 2.5. It is worth noting that this item exhibits the lowest value for physicians (1.67) and the highest for non-physicians (2.37). In addition, physicians are the group attributing the lowest values to postgraduate education and professional experience for acquiring management competencies. In reference to factors of effectiveness, all groups also agree on the necessity of mixing theoretical and practical lessons during the training program with mean values of well above 4.5, whereas exclusive use of self-assessment is generally viewed as the most ineffective practice, except for non-physician, who attribute the lowest value to remote lessons (mean 1.82). Among the challenges, the whole sample and physicians and participants without management positions see the lack of financial support from their organization as the main problem (mean 4.10), while non-physicians and participants with management positions believe this is represented by a lack of time, with mean values, respectively, of 3.75 and 4. All agree that dialogue and discussion during the course have been the least relevant of the problems, with mean values below 1.5. Outcomes show generally high values, as revealed by the fact that the lowest values exhibit mean values around 3.5. It is worth noting that an increased understanding of the healthcare systems has been the main benefit gained from the program, with mean values equal to or higher than 4.50. The lowest positive impact is attributed by all attendees to improved relationships with superiors and top management, with mean values between 3.44 and 3.74, with the exception of participants without management positions who mention improved career prospects.

**Table 2:** Items with the highest and lowest values by group

	**Management competencies before the training program**					
	**Highest**	**Mean**	**SD**	**Lowest**	**Mean**	**SD**
***Sample***	Acquired through professional experience	2.86	1.01	Acquired through university education	1.96	1.07
***Physicians***	Acquired through professional experience	2.80	0.96	Acquired through university education	1.67	0.93
***Non-physicians***	Acquired through postgradutate training programs	3.05	1.22	Acquired through university education	2.37	1.14
***With management position***	Acquired through professional experience	3.17	0.88	Acquired through university education	1.88	0.90
***Without management position***	Acquired through professional experience	2.63	1.04	Acquired through university education	2.01	1.18
	**Factors of effectiveness**					
	**Highest**	**Mean**	**SD**	**Lowest**	**Mean**	**SD**
***Sample***	Theoretical and practical lessons	4.86	0.45	Exclusive use of self-assessment	1.91	1.05
***Physicians***	Theoretical and practical lessons	4.85	0.50	Exclusive use of self-assessment	1.90	1.09
***Non-physicians***	Theoretical and practical lessons	4.88	0.38	Remote lessons	1.82	0.89
***With management position***	Theoretical and practical lessons	4.79	0.59	Exclusive use of self-assessment	2.09	1.08
***Without management position***	Theoretical and practical lessons	4.91	0.32	Exclusive use of self-assessment	1.78	1.02
	**Challenges**					
	**Highest**	**Mean**	**SD**	**Lowest**	**Mean**	**SD**
***Sample***	Lack of financial support from their organization	3.93	1.63	Dialogue and discussion	1.32	0.66
***Physicians***	Lack of financial support from their organization	4.18	1.47	Dialogue and discussion	1.27	0.63
***Non-physicians***	Lack of time	3.75	1.09	Dialogue and discussion	1.40	0.70
***With management position***	Lack of time	4.00	0.97	Dialogue and discussion	1.26	0.52
***Without management position***	Lack of financial support from their organization	4.10	1.54	Dialogue and discussion	1.37	0.75
	**Outcomes**					
	**Highest**	**Mean**	**SD**	**Lowest**	**Mean**	**SD**
***Sample***	Increased understanding of the healthcare systems	4.55	0.83	Improved relationships with superiors and top management	3.49	1.22
***Physicians***	Increased understanding of the healthcare systems	4.58	0.83	Improved relationships with superiors and top management	3.52	1.22
***Non-physicians***	Increased understanding of the healthcare systems	4.51	0.85	Improved relationships with superiors and top management	3.44	1.23
***With management position***	Increased understanding of the healthcare systems	4.50	0.90	Improved relationships with superiors and top management	3.74	1.09
***Without management position***	Increased understanding of the healthcare systems	4.59	0.78	Improved career prospects	3.27	1.33

To shed light on the factors influencing the management training process, the findings of the factor analyses conducted by category are reported. Starting from the management competencies held before the training program, the following single factor was extracted from the four items, named and interpreted as follows:

1. Basic management competencies, which measures the level of management competencies acquired by participants through higher education, post-graduate training and professional experience.

The effectiveness factors are then grouped into six factors, named and explained as follows:

1. Diversity and debate, which aggregates five items assessing the importance of diversity in participants’ and teachers’ educational and professional backgrounds and pedagogical approaches and tools, as well as level of participant engagement and discussion during lessons and in carrying out the project work required to complete the program.
2. Specialization, which includes three items accounting for a robust knowledge of healthcare systems by focusing on teachers’ profiles and lessons’ theoretical approaches.
3. Lessons in presence, which groups three items explaining that in-presence lessons increase learning outcomes and discussion among participants.
4. Final self-assessment, made up of three items asserting that learning outcomes should be assessed by participants themselves at the end of the course.
5. Written intermediate assessment, composed of two items explaining that mid-terms assessment should only be written.
6. Homogeneous class, which is made up of a single component accounting for participants’ similarity in terms of professional backgrounds and management levels, tasks and responsibilities.

The challenges are aggregated into the following four factors:

1. Lack of time, which includes three items reporting scarce time and energy for lessons and study.
2. Problems of dialogue and discussion, which groups three items focusing on difficulties in relating to and debating with other participants and teachers.
3. Low support from organization, which is made up of two items reporting poor financial support and low value given to the initiative from participants’ own organizations.
4. Organizational issues, which aggregates two items demonstrating scarce flexibility and collaboration by superiors and colleagues of participants’ own organizations and unfamiliarity to study.

Table 3 shows the component matrix with saturation coefficients and factors obtained for the management competencies held before the training program (unrotated), effectiveness factors (rotated), and challenges (rotated).

**Table 3:** Factor analyses. Component matrixes

**Management competencies before the training program**	***Basic management competencies***					
Adequate level of management competencies	0.863					
Acquired through university education	0.832					
Acquired through postgraduate training programs	0.887					
Acquired through professional experience	0.765					
**Factors of effectiveness of the training program**	***Diversity and debate***	***Specialization***	***Lessons in presence***	***Final self-assessment***	***Written mid-term assessment***	***Homogeneous class***
Mixed class	**0.766**	0.146	-0.070	-0.091	-0.216	-0.056
Homogeneous class	0.058	0.109	-0.057	0.056	-0.001	**0.916**
Diversity in teaching staff	**0.597**	-0.070	-0.051	-0.123	-0.034	0.430
Teachers from healthcare field	-0.011	**0.798**	0.010	0.101	0.099	0.123
Teachers from academic field	0.069	**0.857**	-0.012	0.016	0.043	0.090
Theoretical lessons	-0.183	**0.622**	-0.099	0.253	-0.150	-0.151
Lessons in presence	0.121	0.184	**0.710**	-0.039	-0.138	0.210
Remote lessons	0.112	0.189	**-0.673**	0.282	-0.011	0.079
Participant engagement and discussion	**0.472**	-0.041	0.334	-0.079	0.252	0.293
Difficulty in discussions during remote lessons	0.036	-0.080	**0.802**	0.056	-0.020	-0.178
Diversity in pedagogical approaches and tools	**0.740**	-0.217	0.207	-0.031	0.103	0.080
Mid-term assessment	0.282	0.079	0.066	**-0.670**	0.159	0.074
Final assessment	0.110	0.187	-0.099	**0.819**	-0.084	0.083
Self-assessment	-0.057	0.352	-0.038	**0.649**	0.304	-0.004
Oral mid-term assessment	-0.036	0.158	0.043	0.173	**-0.814**	-0.100
Written mid-term assessment	0.102	0.381	-0.106	0.154	**0.697**	-0.154
Team project work	**0.752**	0.030	-0.065	0.038	0.179	-0.053
**Challenges**	***Lack of time***	***Problems of dialogue and discussion***	***Low support from organization***	***Organizational issues***		
Lack of time to attend lessons	**0.843**	0.161	0.066	0.123		
Lack of time to study	**0.908**	0.107	0.100	-0.013		
Lack of energy due to workload	**0.848**	-0.021	0.108	0.076		
Scarce flexibility and collaboration by superiors and colleagues	-0.098	0.114	0.417	**0.734**		
Unfamiliarity to study	0.348	0.093	-0.278	**0.734**		
Lack of financial support from own organizations	0.190	-0.125	**0.771**	-0.175		
Low value given to the initiative from own organizations	0.082	0.184	**0.749**	0.248		
Unaccustomed to discussing	0.191	**0.738**	-0.168	0.201		
Problems of dialogue and discussion among participants	0.041	**0.790**	0.07	0.166		
Problems of dialogue and discussion with teachers	0.018	**0.772**	0.121	-0.12		

A set of ordinal logistic regressions was performed to examine the relationships between management competencies held before the start of the course, effectiveness factors, challenges and outcomes of the training program. The results, shown in Table 4, are articulated into four models, one for each selected outcome. In relation to model 1, the factors ‘diversity and debate’ (*p* < 0.001), ‘written intermediate assessment’ (*p* < 0.05) and ‘homogeneous class’ (*p* < 0.001) have a significant positive impact on the improvement of management competencies, which is also increased by low values attributed to ‘problems of dialogue and discussion’ (*p* < 0.01). In model 2, the change of professional practices in light of lessons learned during the program, selected as an innovation outcome, is then positively affected by ‘diversity and debate’ (*p* < 0.001), ‘homogeneous class’ (*p* < 0.05) and ‘organizational issues’ (*p* < 0.01), while it was negatively influenced by a high value of ‘basic management competencies’ held before the course (*p* < 0.05). Regarding model 3, ‘Diversity and debate’ (*p* < 0.001) and ‘homogeneous class’ (*p* < 0.01) have a significant positive effect on the improvement of professional relationships as well, whereas the same is negatively affected by ‘lessons in presence’ (*p* < 0.05). Finally, concerning model 4, the outcome career prospects benefit from ‘diversity and debate’ (*p* < 0.05) and ‘homogeneous class’ (*p* < 0.01), since both factors exert a positive effect. ‘Low support from organization’ negatively influences career prospects (*p* < 0.001). Table 4 also shows that the LR test of proportionality of odds across the response categories cannot be rejected (all four *p* > 0.05).

**Table 4:** Relating outcomes to managerial competencies, factors of effectiveness and challenges of the training program

	**Ordered logit models for outcomes**							
	*Model 1*		*Model 2*		*Model 3*		*Model 4*	
**Independent variables**	***Managerial competencies***		***Innovation practices***		***Professional relationships***		***Career prospects***	
	Coeff	Z score	Coeff	Z score	Coeff	Z score	Coeff	Z score
Managerial competencies held before			-.407*	-2.38				
Diversity and debate	1.298***	6.21	.742***	4.54	.606***	3.76	.393*	2.51
Specialization			.252	1.49				
Lessons in presence					-.365*	-2.15		
Final self-assessment	.294	1.39						
Written mid-term assessment	.505*	2.52						
Homogeneous class	.827***	3.90	.445*	2.56	.475**	2.84	.506**	3.13
Problems of dialogue and discussion	-.623**	-3.09						
Low support from organization	.325	1.65					-.579***	-3.55
Organizational issues	.356	1.62	.476**	2.75				
*Model*								
Observations	140		140		140		140	
Pseudo R^2^	0.26 (0.000)		0.10 (0.000)		0.06 (0.000)		0.07 (0.000)	
LR test of POA (x^2^)	25.59		15.01		6.31		8.97	
LR test of POA (*p*)	0.22		0.45		0.70		0.44	

*POA *Proportional Odds Assumption (null-hypothesis is that there is no violation of this assumption)

* p < 0.05

** p < 0.01

*** p < 0.001 (two-tailed)

Finally, it is worth noting that none of the control variables reflecting the respondent profiles (age, gender, management position, working in the healthcare field, and educational background) was found to be statistically significant. These variables are not reported in Table 4 because regression models were obtained following a backward elimination method, as explained in the method section.

In the end, the t-test reveals significant differences between physicians and non-physicians, as well as between participants with or without management positions. Table 5 shows only figures of t-test statistically significant with regards to competencies held before the attendance of the course, the factors of effectiveness, challenges of the training program, and outcomes achieved. In the first comparison, non-physicians show higher management competencies at the start of the program, with a mean value of 0.31, while physicians suffer from less support from their own organization with a mean value of 0.13 compared to -0.18, the mean value of the non-physicians. Concerning the second comparison, participants with management positions have higher management competencies at the start of the program (0.19 versus -0.13) and suffer more from lack of time, with higher mean values compared to participants without managerial positions, respectively 0.23 and -0.16. For what concerns the factors related to the effectiveness of the training program, participants with management positions exhibit a lower mean value in relation to written mid-term assessments, -0.24 versus 0.17, reported by participants with management positions. Differently, the final self-assessment at the end of the program is higher for participants with management positions, 0.24 compared to -0.17, the mean value of the participants without management positions. This latter category feels more the problem of low support from their organizations, with a mean value of 0.16 compared to -0.23, and is slightly less motivated by possible career improvement, with a mean value of 3.31 compared to 3.73 reported by participants with management positions.

**Table 5:** Unpaired two-sample t-test

	***Mean***		***Significance***
***Variables***	*Physicians*	*Non-physicians*	
Managerial competencies held before	-.22	.31	0.02
Low support from organization	.13	-.18	0.074
	*With management position*	*Without management position*	
Managerial competencies held before	.19	-.13	0.052
Final self-assessment	.24	-.17	0.018
Written mid-term assessment	-.24	.17	0.017
Lack of time	.23	-.16	0.016
Low support from organization	-.23	.16	0.022
Career prospects	3.73	3.31	0.054

## Discussion

The results stemming from the different analyses are now considered and interpreted in the light of the extant literature. Personal characteristics such as gender and age, differently from what was found by Walston et al. [26] for executives’ continuing education, and professional characteristics such as seniority and working in public or private sectors, do not seem to affect participation in management training programs.

The findings clearly show the outstanding importance of ‘diversity and debate’ and ‘class homogeneity’ as factors of effectiveness, since they positively impact all outcomes: competencies, innovation, professional relationships and career. These factors capture two key aspects complementing each other: on the one hand, participants and teachers’ different backgrounds provide the class with a wider pool of resources and expertise, whereas the use of pedagogical tools fostering discussion enriches the educational experience and stimulates creativity. On the other hand, due to the high level of professionalism in the setting, sharing common management levels means similar tasks and responsibilities, as well as facing similar problems. Consequently, speaking the same language leads to deeper knowledge and effective technical solutions.

In relation to the improvement of management competencies, it also emerges the critical role of a good class atmosphere, that is, the absence of problems of dialogue and discussion. ‘Diversity and debate’ and ‘class homogeneity’, as explained before, seem to contribute to this, since they enhance freedom of expression and fair confrontation, leading to improved learning outcomes. It is interesting to notice that the problems of dialogue and discussion turned out to be the least relevant challenge across the sample.

Two interesting points come from the factors affecting innovation. First, it seems that lower competencies before the training programs lead to the development of more innovative practices. The reason is that holding fewer basic competencies means a greater scope for action once new capabilities are learned: the spirit of openness is conducive to breaking down routines, and innovative practices hindered by a lack of knowledge and tools can thus be introduced. The reason is that holding fewer basic competencies means greater scope for action once new capabilities are learned: the spirit of openness is conducive to breaking down routines, and innovative practices hindered by a lack of knowledge and tools can thus be introduced. This extends the findings of previous studies since the employment of competencies in the workplace is influenced by the starting competence equipment of professionals [36], and those showing gaps have more room to recover, also in terms of motivation to change, that is, understanding the importance of meeting current and future challenges [26]. Second, more innovative practices are introduced by participants perceiving more organizational issues. This may reveal, on the one side, a stronger individual motivation towards professional growth of participants who suffer from lack of flexibility and collaboration from their own superiors and colleagues. In this regard, poor tolerance, flexibility and permissions in their workplace act as a stimulus to innovation, which can be viewed as a way of challenging the status quo. On the other side, in line with the above-mentioned concept, this confirms that unfamiliarity with the study increases the innovative potential of participants. Since this study reveals that physicians are neither adequately educated from a management point of view nor incentivized to attend post-graduation training programs, it points out how important is extending continuing education to all health professional categories [25, 26].

The topic of competencies held by different categories needs more attention. The study reveals that physicians and participants without management positions start the program with less basic competencies. At the sample level, higher education is viewed as the most ineffective tool to provide such competencies, whereas professional experience is seen as the best way to gather them. Actually, non-physicians give the highest value to postgraduate education, which suggests they are those more interested or incentivized to take part in continuing education. Although holding managerial positions does not automatically mean having higher competencies [5], it is evident that such a professional experience contributes to filling existing gaps. Physicians stand out as the category for which university education, postgraduate education and professional experience exert the lowest impact on management competence improvement. Considering the relationship between competence held before the course and innovation, as described above, engaging physicians in training programs, even more if they do not have management responsibilities, has a major impact on health organizations’ development prospects. The findings also point out that effective management training requires a combination of theory and practice for all categories of professionals, not just for physicians, as observed by Loh [5].

The main outcome, in general and for all participant categories, is an increased understanding of how healthcare systems work, which anticipates increased competencies. This confirms the importance of knowledge on the healthcare environment [31], and clarifies the order of aspects impacted by training programs as reported by Ravaghi et al. [36]: first conceptual, then technical, and finally interpersonal. However, interpersonal outcomes are by far greater for those holding management positions, which extends the findings by Liang et al. [31]. In particular, participants already managing units report the greatest impacts in terms of ability to understand colleagues’ problems, improvement of professional relationships and collaboration with colleagues from other units. Obviously, participants with management positions, more than others, feel the lack of collaborative and communication skills, which represents one of the main flaws of university education in the field of medicine [4] and is also often neglected in management training [36]. This also confirms that different management levels show specific competence requirements and education needs [6, 7]. 

It is then important to discuss the negative effect of lessons in presence on the improvement of professional relationships. At first glance, it may sound strange, but its real meaning emerges from a comprehensive interpretation of all the findings. First, it does not mean that remote lessons are more effective, as revealed by the fact that they, as a factor of effectiveness, are attributed very low values and, for all categories of participants, lower values than those attributed to lessons in presence and hybrid lessons. Non-physicians, in particular, attribute them the lowest value at all. At most, remote lessons are viewed as convenient rather than effective. The negative influence of lessons in presence can be explained by the fact that a specific category, i.e., those with management positions, rate this aspect much more important than other participants and, as reported above, find much more benefits in terms of improved relationships from management training. Participants with management positions, due to their tasks and responsibilities, suffer more than others from lack of time to be devoted to course participation. For them, as for the category of non-physicians, lack of time represents the main challenge to effectively attending the course. In the literature, such a problem is well considered, and lack of time is also viewed as a challenge to apply the skills learned during the course [36]. Considering that class discussion and homogeneity contribute to fostering relationships, a comprehensive reading of the findings reveals that due to workload, participants with management positions see particularly convenient and still effective remote lessons. Furthermore, if the class is formed by participants sharing similar professional backgrounds and management levels, debate is not precluded and interpersonal relationships improved as a consequence. From the observation of single items, it can be concluded that participants with management positions and in general those with higher basic management competencies at the start of the program, prefer more flexible and leaner training programs: intermediate assessment through conversation, self-assessment at the end of the course, more concentrated scheduled lessons and greater use of remote lessons.

Differently from what was found by Walston and Khaliq [25], the findings highlight that participants with management positions value the impact of management training on career prospects positively. These participants are also those more supported by their own organizations. Conversely, the lack of support, especially in terms of inadequate funds devoted to these initiatives, strongly affects physicians and participants without management positions, which clarifies what this challenge is about and who is mainly affected by it [36]. Low incentives mean having attended fewer training programs in the past, which, together with less management experience, explains why they have developed less competencies. Among the outcomes of the training program, the little attention paid by organizations is also testified by the lowest values attributed by all categories, except for participants without management positions, to the improvement of relationships with superiors and top management.

## Conclusions

In general, the study contributes to a better understanding of the outcomes of management training programs in healthcare and their determinants [9]. In particular, it sheds light on gaps and education needs [1] by category of health professionals [2]. The research findings have major implications for practice, which can be drawn after identifying the four profiles of participants revealed by the study. All profiles share common characteristics, such as value given to debate, diversity of pedagogical approaches and tools and class homogeneity, rather than the need for a deeper comprehension of healthcare systems. However, they present characteristics that determine specific issues and education gaps, which are summarized as follows:

1. Physicians without management positions: low competencies at the start of the program and scarce incentives for attending the course from their own organization;
2. Physicians with management positions: they partially compensate for competence gaps through professional experience, suffer from lack of time, and are motivated by the chance to improve their career prospects;
3. Non-physicians without management positions: they partially fill competence gaps through postgraduate education, suffer from lack of time, and have scarce incentives for attending the course from their own organization;
4. Non-physicians with management positions: they partially bridge competence gaps through postgraduate education and professional experience, are the most affected by a lack of time, and are motivated by the chance to improve their career prospects.

Recommendations are outlined for different levels of action:

- For policymakers, it is suggested to strengthen the ability of higher education courses in medicine and related fields to advance the understanding of healthcare systems’ structure and operation, as well as their current and future challenges. Such a new approach in the design curricula should then have as a main goal the provision of adequate management competencies.
- For healthcare organizations, it is suggested to incentivize the acquisition of management competencies by all categories of professionals through postgraduate education and training programs. This means supporting them from both financial and organizational point of view, for instance, in terms of more flexible working conditions. Special attention should be paid to physicians who, even without executive roles, manage resources and directly impact the organization's effectiveness and efficiency levels through their day-by-day activity, and are the players holding the greatest innovative potential within the organization. Concerning the executives, especially in the current changing context of healthcare systems, much higher attention should be paid to fostering interpersonal skills, in terms of communication and cooperation.
- For those designing training programs, it is suggested to tailor courses on the basis of participants’ profiles, using different pedagogical approaches and tools, for instance, in terms of teacher composition, lesson delivery methods and learning assessment methods, while preserving class homogeneity in terms of professional backgrounds and management levels to facilitate constructive dialogue and solution finding approaches. Designing ad hoc training programs would give the possibility to meet the needs of participants from an organizational point of view as well as, for instance, in terms of program length and lesson concentration.

### Limitations

This study has some limitations, which pave the way for future research. First, it is context-specific by country, since it is carried out within the INHS, which mandatorily requires health professionals to attend management training programs to hold certain positions. It is then context-specific by training program, since it focuses on management training programs providing participants with the title to be appointed as a director of a ward or administrative unit in a public healthcare organization. This determines the kind of management competencies included in the study, which are those mandatorily required for such a middle-management category. Therefore, there is a need to extend research and test these findings on different types of management training programs, participants and countries. Second, this study is based on a survey of participants’ perceptions, which causes two kinds of unavoidable issues: although based on the literature and pre-tested, the questionnaire could not be able to measure what it intends to or capture detailed and nuanced insights from respondents, and responses may be affected by biases due to reactive effects. Third, a backward elimination method was adopted to select variables in model building. Providing a balance between simplicity and fit of models, this variable selection technique is not consequences-free. Despite advantages such as starting the process with all variables included, removing the least important early, and leaving the most important in, it also has some disadvantages. The major is that once a variable is deleted from the model, it is not included anymore, although it may become significant later [38]. For these reasons, it is intended to reinforce research with new data sources, such as teachers’ perspectives and official assessments, and different variable selection strategies. A combination of qualitative and quantitative methods for data elaboration could then be used to deepen the analysis of the relationships between motivations, effectiveness factors and outcomes. Furthermore, since the investigation of competence development, acquisition of new competencies and the transfer of acquired competencies was beyond the purpose of this study, a longitudinal approach will be used to collect data from participants attending future training programs to track changes and identify patterns.

### Supplementary Information

Supplementary Material 1.

## Data Availability

An English-language version of the questionnaire used in this study is attached to this paper as a supplementary file. The raw data collected via the questionnaire are not publicly available due to privacy and other restrictions. However, datasets generated and analyzed during the current study may be available from the corresponding author upon reasonable request.

## Abbreviations

INHS: Italian National Health System
KMO: Kaiser–Meyer–Olkin
NRRP: National Recovery and Resilience Plan

## References

[1] Liang Z, Howard PF, Wang J, Xu M, Zhao M. Developing senior hospital managers: does ‘one size fit all’? – evidence from the evolving Chinese health system. BMC Health Serv Res. 2020;20(281):1–14. 10.1186/s12913-020-05116-6. 10.1186/s12913-020-05116-6 PMC7137490 32252749

[2] Kakemam E, Liang Z. Guidance for management competency identification and development in the health context: a systematic scoping review. BMC Health Serv Res. 2023;23(421):1–13. 10.1186/s12913-023-09404-9. 37127614 10.1186/s12913-023-09404-9 PMC10150671

[3] European Commission. Annual Sustainable Growth Strategy. 2020. https://eur-lex.europa.eu/legal-content/EN/TXT/PDF/?uri=CELEX:52020DC0575&from=en

[4] Blakeney EAR, Ali HN, Summerside N. Sustaining improvements in relational coordination following team training and practice change: a longitudinal analysis. Health Care Manag Rev. 2021;46(4):349–57. 10.1097/HMR.0000000000000288. 10.1097/HMR.0000000000000288 PMC7775290 32649474

[5] Loh E. How and why medically-trained managers undertake postgraduate management training - a qualitative study from Victoria. J Health Organ Manag. 2015;29(4):438–54. 10.1108/jhom-10-2013-0233. 26045189 10.1108/jhom-10-2013-0233

[6] Yarbrough LA, Stowe M, Haefner J. Competency assessment and development among health-care leaders: results of a cross-sectional survey. Health Serv Manag Res. 2012;25(2):78–86. 10.1258/hsmr.2012.012012. 10.1258/hsmr.2012.012012 22673697

[7] Liang Z, Blackstock FC, Howard PF, Briggs DS, Leggat SG, Wollersheim D, Edvardsson D, Rahman A. An evidence-based approach to understanding the competency development needs of the health service management workforce in Australia. BMC Health Serv Res. 2018;18(976):1–12. 10.1186/s12913-018-3760-z. 30563505 10.1186/s12913-018-3760-z PMC6299513

[8] Whaley A, Gillis WE. Leadership development programs for health care middle managers: an exploration of the top management team member perspective. Health Care Manag Rev. 2018;43(1):79–89. 10.1097/HMR.0000000000000131. 10.1097/HMR.0000000000000131 27755175

[9] Campbell C, Lomperis A, Gillespie K, Arrington B. Competency-based healthcare management education: the Saint Louise University experience. J Health Adm Educ. 2006;23:135–68. 16700441

[10] Issel ML. Value Added of Management to Health Care Organizations. Health Care Manag Rev. 2020;45(2):95. 10.1097/HMR.0000000000000280. 10.1097/HMR.0000000000000280 32106118

[11] Lega F, Prenestini A, Spurgeon P. Is management essential to improving the performance and sustainability of health care systems and organizations? a systematic review and a roadmap for future studies. Value Health. 2013;16(1 Suppl.):S46–51. 10.1016/j.jval.2012.10.004. 23317645 10.1016/j.jval.2012.10.004

[12] Bloom N, Propper C, Seiler S, Van Reenen J. Management practices in hospitals. Health, Econometrics and Data Group (HEDG) working papers 09/23, HEDG, c/o department of economics, University of York. 2009.

[13] Vainieri M, Ferrè F, Giacomelli G, Nuti S. Explaining performance in health care: how and when top management competencies make the difference. Health Care Manag Rev. 2019;44(4):306–17. 10.1097/HMR.0000000000000164. 10.1097/HMR.0000000000000164 PMC6749958 28448307

[14] Del Vecchio M, Carbone C. Stabilità dei Direttori Generali nelle aziende sanitarie. In: Anessi Pessina E, Cantù E, editors. Rapporto OASI 2002 L’aziendalizzazione della sanità in Italia. Milano, Italy: Egea; 2002. p. 268–301.

[15] McAlearney AS. Leadership development in healthcare: a qualitative study. J Organ Behav. 2006;27:967–82. 10.1002/job.417

[16] McAlearney AS. Using leadership development pro- grams to improve quality and efficiency in healthcare. J Healthcare Manag. 2008;53:319–31. 10.1097/00115514-200809000-00008 18856137

[17] McAlearney AS. Executive leadership development in U.S. health systems. J Healthcare Manag. 2010;55:207–22. 10.1097/00115514-201005000-00011 20565036

[18] McAlearney AS, Fisher D, Heiser K, Robbins D, Kelleher K. Developing effective physician leaders: changing cultures and transforming organizations. Hosp Top. 2005;83(2):11–8. 16190516

[19] Thompson JM, Kim TH. A profile of hospitals with leadership development programs. Health Care Manag. 2013;32(2):179–88. 10.1097/HCM.0b013e31828ef677. 10.1097/HCM.0b013e31828ef677 23629041

[20] Figueroa C, Harrison R, Chauhan A, Meyer L. Priorities and challenges for health leadership and workforce management globally: a rapid review. BMC Health Serv Res. 2019;19(239):1–11. 10.1186/s12913-019-4080-7. 31014349 10.1186/s12913-019-4080-7 PMC6480808

[21] Sarto F, Veronesi G. Clinical leadership and hospital performance: assessing the evidence base. BMC Health Serv Res. 2016;16(169):85–109. 10.1186/s12913-016-1395-5. 27230873 10.1186/s12913-016-1395-5 PMC4896259

[22] Morandi F, Angelozzi D, Di Vincenzo F. Individual and job-related determinants of bias in performance appraisal: the case of middle management in health care organizations. Health Care Manag Rev. 2021;46(4):299–307. 10.1097/HMR.0000000000000268. 10.1097/HMR.0000000000000268 31855878

[23] Tasi MC, Keswani A, Bozic KJ. Does physician leadership affect hospital quality, operational efficiency, and financial performance? Health Care Manag Rev. 2019;44(3):256–62. 10.1097/hmr.0000000000000173. 10.1097/hmr.0000000000000173 28700509

[24] Hopkins J, Fassiotto M, Ku MC. Designing a physician leadership development program based on effective models of physician education. Health Care Manag Rev. 2018;43(4):293–302. 10.1097/HMR.0000000000000146. 10.1097/HMR.0000000000000146 PMC5540850 28157830

[25] Walston SL, Khaliq AA. The importance and use of continuing education: findings of a national survey of hospital executives. J Health Admin Educ. 2010;27(2):113–25.

[26] Walston SL, Chou AF, Khaliq AA. Factors affecting the continuing education of hospital CEOs and their senior managers. J Healthcare Manag. 2010;55(6):413–27. 10.1097/00115514-201011000-00008. 10.1097/00115514-201011000-00008 21166324

[27] Garman AN, McAlearney AS, Harrison MI, Song PH, McHugh M. High-performance work systems in health- care management, part 1: development of an evidence-informed model. Health Care Manag Rev. 2011;36(3):201–13. 10.1097/HMR.0b013e318201d1bf. 10.1097/HMR.0b013e318201d1bf 21646880

[28] MacDavitt K, Chou S, Stone P. Organizational climate and healthcare outcomes. Joint Comm J Qual Patient Saf. 2007;33(S11):45–56. 10.1016/s1553-7250(07)33112-7. 10.1016/s1553-7250(07)33112-7 18173165

[29] Singer SJ, Hayes J, Cooper JB, Vogt JW, Sales M, Aristidou A, Gray GC, Kiang MV, Meyer GS. A case for safety leadership training of hospital manager. Health Care Manag Rev. 2011;36(2):188–200. 10.1097/HMR.0b013e318208cd1d. 10.1097/HMR.0b013e318208cd1d 21317660

[30] Liang Z, Leggat SG, Howard PF, Lee K. What makes a hospital manager competent at the middle and senior levels? Aust Health Rev. 2013;37(5):566–73. 10.1071/AH12004. 23601561 10.1071/AH12004

[31] Liang Z, Howard PF, Koh L, Leggat SG. Competency requirements for middle and senior managers in community health services. Aust J Prim Health. 2013;19(3):256–63. 10.1071/PY12041. 23007275 10.1071/PY12041

[32] AbuDagga A, Weech-Maldonado R, Tian F. Organizational characteristics associated with the provision of cultural competency training in home and hospice care agencies. Health Care Manag Rev. 2018;43(4):328–37. 10.1097/HMR.0000000000000144. 10.1097/HMR.0000000000000144 PMC5472501 27984407

[33] Liang Z, Howard PF, Leggat SG, Bartram T. Development and validation of health service management competencies. J Health Organ Manag. 2018;32(2):157–75. 10.1108/JHOM-06-2017-0120. (Epub 2018 Feb 8). 29624143 10.1108/JHOM-06-2017-0120

[34] Howard PF, Liang Z, Leggat SG, Karimi L. Validation of a management competency assessment tool for health service managers. J Health Organ Manag. 2018;32(1):113–34. 10.1108/JHOM-08-2017-0223. 29508674 10.1108/JHOM-08-2017-0223

[35] Fanelli S, Lanza G, Enna C, Zangrandi A. Managerial competences in public organisations: the healthcare professionals’ perspective. BMC Health Serv Res. 2020;20(303):1–9. 10.1186/s12913-020-05179-5. 10.1186/s12913-020-05179-5 PMC7158078 32293450

[36] Ravaghi H, Beyranvand T, Mannion R, Alijanzadeh M, Aryankhesal A, Belorgeot VD. Effectiveness of training and educational programs for hospital managers: a systematic review. Health Serv Manag Res. 2020;34(2):1–14. 10.1177/0951484820971460. 10.1177/0951484820971460 33143488

[37] Woltring C, Constantine W, Schwarte L. Does leadership training make a difference? J Public Health Manag Prac. 2003;9(2):103–22. 10.1097/00124784-200303000-00004 12629912

[38] Chowdhury MZI, Turin TC. Variable selection strategies and its importance in clinical prediction modelling. Fam Med Comm Health. 2020;8(1):1–7. 10.1136/fmch-2019-000262. 10.1136/fmch-2019-000262 PMC7032893 32148735

