# Supplementary material for: Management training programs in healthcare: effectiveness factors, challenges and outcomes
Source: BMC Health Serv Res. 2024 Aug 7;24:904. doi: 10.1186/s12913-024-11229-z (PMC11308623; doi:10.1186/s12913-024-11229-z)
Supplement: Supplementary file 1 — Supplementary Material 1. [file 12913_2024_11229_MOESM1_ESM.docx]

**Questionnaire: Management training programs in healthcare: factors of effectiveness, challenges and outcomes**

Section 1: Participant profile

1. Gender
   - Male
   - Female
   - I prefer not to say
2. Age
   - Between 20 and 29 years
   - Between 30 and 39 years
   - Between 40 and 49 years
   - Between 50 and 59 years
   - Over 60 years
3. Educational background
   - Medicine
   - Health professions
   - Economics
   - Law
   - Engineering
   - Pharmacy
   - Chemistry
   - Veterinary medicine
   - Other
4. Before starting the program, have you ever managed health organizations/structures?
   - Yes
   - No
5. Are you currently holding management roles in health organizations/structures?
   - Yes
   - No
6. Are you currently working in health organizations/structures?
   - Yes
   - No
7. If you answered ‘Yes’ to the previous question, is the health organization in which you work public?
   - Yes
   - No
8. How long have you worked?

Working years: __________

1. Why did you choose to attend the program?
   - I was obliged
   - To improve my career prospects
   - To improve my competencies
   - To improve my professional relationships
   - To emulate colleagues who attended it before
   - I was incentivized by my organization
   - Other
2. If you answered ‘Other’ to the previous question, please specify: __________________________________________________________________________

Section 2: Management competencies held by participants before the training program

*This section aims to shed light on the participants’ situation regarding management competencies held before the start of the training program. It is clarified that ‘Basic management competencies’, in line with Presidential Decree No. 484/1997, include knowledge and skills related to the organization and management of health care services, funding models and accounting issues, human resource management and performance measurement. Respondents must express their opinion on each item using a scale of values ranging from 1 (Completely disagree) to 5 (Completely agree).*

1. My basic management competencies were adequate:
   - 1 (Completely disagree)
   - 2 (Mostly agree)
   - 3 (Undecided)
   - 4 (Mostly disagree)
   - 5 (Completely agree)
2. University education permitted me to develop adequate management competencies:
   - 1 (Completely disagree)
   - 2 (Mostly agree)
   - 3 (Undecided)
   - 4 (Mostly disagree)
   - 5 (Completely agree)
3. Postgraduate training programs permitted me to develop adequate management competencies:
   - 1 (Completely disagree)
   - 2 (Mostly agree)
   - 3 (Undecided)
   - 4 (Mostly disagree)
   - 5 (Completely agree)
4. I autonomously developed, through professional experience, adequate management competencies:
   - 1 (Completely disagree)
   - 2 (Mostly agree)
   - 3 (Undecided)
   - 4 (Mostly disagree)
   - 5 (Completely agree)

Section 3: Factors of effectiveness of the training program

*This section aims to collect participants’ opinions regarding the ways in which the program was conducted and the factors influencing its effectiveness. Based on their personal experience, respondents must express their opinion on each item using a scale of values ranging from 1 (Completely disagree) to 5 (Completely agree).*

1. Mixed class composition (in terms of professional backgrounds and management levels, tasks and responsibilities) fosters discussion and enhances learning:
   - 1 (Completely disagree)
   - 2 (Mostly agree)
   - 3 (Undecided)
   - 4 (Mostly disagree)
   - 5 (Completely agree)
2. Homogeneous class composition (in terms of professional backgrounds and management levels, tasks and responsibilities) fosters discussion and enhances learning:
   - 1 (Completely disagree)
   - 2 (Mostly agree)
   - 3 (Undecided)
   - 4 (Mostly disagree)
   - 5 (Completely agree)
3. Mixed teacher staff provides multiple perspectives that enhance learning:
   - 1 (Completely disagree)
   - 2 (Mostly agree)
   - 3 (Undecided)
   - 4 (Mostly disagree)
   - 5 (Completely agree)
4. Teachers should all come from the healthcare field:
   - 1 (Completely disagree)
   - 2 (Mostly agree)
   - 3 (Undecided)
   - 4 (Mostly disagree)
   - 5 (Completely agree)
5. Teachers should all come from the academic field:
   - 1 (Completely disagree)
   - 2 (Mostly agree)
   - 3 (Undecided)
   - 4 (Mostly disagree)
   - 5 (Completely agree)
6. Lessons should only be theoretical:
   - 1 (Completely disagree)
   - 2 (Mostly agree)
   - 3 (Undecided)
   - 4 (Mostly disagree)
   - 5 (Completely agree)
7. Lessons should only be practical:
   - 1 (Completely disagree)
   - 2 (Mostly agree)
   - 3 (Undecided)
   - 4 (Mostly disagree)
   - 5 (Completely agree)
8. Lessons should only be both theoretical and practical:
   - 1 (Completely disagree)
   - 2 (Mostly agree)
   - 3 (Undecided)
   - 4 (Mostly disagree)
   - 5 (Completely agree)
9. Lessons in presence are more effective:
   - 1 (Completely disagree)
   - 2 (Mostly agree)
   - 3 (Undecided)
   - 4 (Mostly disagree)
   - 5 (Completely agree)
10. Remote lessons are more effective:
    - 1 (Completely disagree)
    - 2 (Mostly agree)
    - 3 (Undecided)
    - 4 (Mostly disagree)
    - 5 (Completely agree)
11. Remote lessons are more convenient:
    - 1 (Completely disagree)
    - 2 (Mostly agree)
    - 3 (Undecided)
    - 4 (Mostly disagree)
    - 5 (Completely agree)
12. Mixed lessons (in presence and remote) are more effective:
    - 1 (Completely disagree)
    - 2 (Mostly agree)
    - 3 (Undecided)
    - 4 (Mostly disagree)
    - 5 (Completely agree)
13. Participant engagement and discussion enhance learning:
    - 1 (Completely disagree)
    - 2 (Mostly agree)
    - 3 (Undecided)
    - 4 (Mostly disagree)
    - 5 (Completely agree)
14. Participant engagement and discussion are more difficult with remote lessons:
    - 1 (Completely disagree)
    - 2 (Mostly agree)
    - 3 (Undecided)
    - 4 (Mostly disagree)
    - 5 (Completely agree)
15. Diversity in pedagogical approaches and tools (theoretical and practical lessons, business games, witness testimonies, case studies, exercises, teamwork) enhances learning:
    - 1 (Completely disagree)
    - 2 (Mostly agree)
    - 3 (Undecided)
    - 4 (Mostly disagree)
    - 5 (Completely agree)
16. Concentrated scheduled lessons enhance learning:
    - 1 (Completely disagree)
    - 2 (Mostly agree)
    - 3 (Undecided)
    - 4 (Mostly disagree)
    - 5 (Completely agree)
17. Scheduled lessons more diluted over time enhance learning:
    - 1 (Completely disagree)
    - 2 (Mostly agree)
    - 3 (Undecided)
    - 4 (Mostly disagree)
    - 5 (Completely agree)
18. Mid-term assessment of competencies acquired during the course is important:
    - 1 (Completely disagree)
    - 2 (Mostly agree)
    - 3 (Undecided)
    - 4 (Mostly disagree)
    - 5 (Completely agree)
19. A final assessment of competencies acquired during the course should only be performed:
    - 1 (Completely disagree)
    - 2 (Mostly agree)
    - 3 (Undecided)
    - 4 (Mostly disagree)
    - 5 (Completely agree)
20. Self-assessment of competencies acquired during the course is sufficient:
    - 1 (Completely disagree)
    - 2 (Mostly agree)
    - 3 (Undecided)
    - 4 (Mostly disagree)
    - 5 (Completely agree)
21. Mid-term assessment of competencies acquired during the course should only be oral:
    - 1 (Completely disagree)
    - 2 (Mostly agree)
    - 3 (Undecided)
    - 4 (Mostly disagree)
    - 5 (Completely agree)
22. Mid-term assessment of competencies acquired during the course should only be written:
    - 1 (Completely disagree)
    - 2 (Mostly agree)
    - 3 (Undecided)
    - 4 (Mostly disagree)
    - 5 (Completely agree)
23. A team project work to complete the program is important to turn acquired competencies into practical solutions:
    - 1 (Completely disagree)
    - 2 (Mostly agree)
    - 3 (Undecided)
    - 4 (Mostly disagree)
    - 5 (Completely agree)

Section 4: Challenges

*This section aims to collect participants’ opinions regarding the main obstacles encountered during the program. Based on their personal experience, respondents must express their opinion on each item using a scale of values ranging from 1 (Completely disagree) to 5 (Completely agree).*

1. It was hard to find time to attend the lessons:
   - 1 (Completely disagree)
   - 2 (Mostly agree)
   - 3 (Undecided)
   - 4 (Mostly disagree)
   - 5 (Completely agree)
2. It was hard to find time to study:
   - 1 (Completely disagree)
   - 2 (Mostly agree)
   - 3 (Undecided)
   - 4 (Mostly disagree)
   - 5 (Completely agree)
3. Due to my workload, it was hard to find the energy required to attend the lessons and study:
   - 1 (Completely disagree)
   - 2 (Mostly agree)
   - 3 (Undecided)
   - 4 (Mostly disagree)
   - 5 (Completely agree)
4. I found scarce flexibility and collaboration (working hours, permissions and support) by superiors and colleagues from my organization:
   - 1 (Completely disagree)
   - 2 (Mostly agree)
   - 3 (Undecided)
   - 4 (Mostly disagree)
   - 5 (Completely agree)
5. I suffer from unfamiliarity with studying:
   - 1 (Completely disagree)
   - 2 (Mostly agree)
   - 3 (Undecided)
   - 4 (Mostly disagree)
   - 5 (Completely agree)
6. I suffer from a lack of financial support from my organization:
   - 1 (Completely disagree)
   - 2 (Mostly agree)
   - 3 (Undecided)
   - 4 (Mostly disagree)
   - 5 (Completely agree)
7. I found that a low value was given to the initiative from my organization:
   - 1 (Completely disagree)
   - 2 (Mostly agree)
   - 3 (Undecided)
   - 4 (Mostly disagree)
   - 5 (Completely agree)
8. I found difficulties because I was unaccustomed to discussing (anxiety, embarrassment and reticence):
   - 1 (Completely disagree)
   - 2 (Mostly agree)
   - 3 (Undecided)
   - 4 (Mostly disagree)
   - 5 (Completely agree)
9. I had problems of dialogue and discussion with the other participants:
   - 1 (Completely disagree)
   - 2 (Mostly agree)
   - 3 (Undecided)
   - 4 (Mostly disagree)
   - 5 (Completely agree)
10. I had problems of dialogue and discussion with the teachers:
    - 1 (Completely disagree)
    - 2 (Mostly agree)
    - 3 (Undecided)
    - 4 (Mostly disagree)
    - 5 (Completely agree)

Section 5: Outcomes

*This section aims to reveal the main outcomes of the program, in terms of knowledge, skills, practices and career. Based on their personal experience, respondents must express their opinion on each item using a scale of values ranging from 1 (Completely disagree) to 5 (Completely agree).*

1. Attending the training program improved my management competencies:
   - 1 (Completely disagree)
   - 2 (Mostly agree)
   - 3 (Undecided)
   - 4 (Mostly disagree)
   - 5 (Completely agree)
2. Attending the training program increased my understanding of healthcare systems:
   - 1 (Completely disagree)
   - 2 (Mostly agree)
   - 3 (Undecided)
   - 4 (Mostly disagree)
   - 5 (Completely agree)
3. Attending the training program increased my understanding of my organization’s problems:
   - 1 (Completely disagree)
   - 2 (Mostly agree)
   - 3 (Undecided)
   - 4 (Mostly disagree)
   - 5 (Completely agree)
4. Attending the training program helped me to see my job in a new way (responsibilities, functions, relationships):
   - 1 (Completely disagree)
   - 2 (Mostly agree)
   - 3 (Undecided)
   - 4 (Mostly disagree)
   - 5 (Completely agree)
5. In the light of lessons learned, I modified some aspects of my job:
   - 1 (Completely disagree)
   - 2 (Mostly agree)
   - 3 (Undecided)
   - 4 (Mostly disagree)
   - 5 (Completely agree)
6. In the light of lessons learned, I introduced innovative practices in my job:
   - 1 (Completely disagree)
   - 2 (Mostly agree)
   - 3 (Undecided)
   - 4 (Mostly disagree)
   - 5 (Completely agree)
7. Attending the training program helped me to understand the problems of people working with me (same structure) better:
   - 1 (Completely disagree)
   - 2 (Mostly agree)
   - 3 (Undecided)
   - 4 (Mostly disagree)
   - 5 (Completely agree)
8. Attending the training program helped me to understand the problems of people working around me (other structures) better:
   - 1 (Completely disagree)
   - 2 (Mostly agree)
   - 3 (Undecided)
   - 4 (Mostly disagree)
   - 5 (Completely agree)
9. Attending the training program improved my professional relationships:
   - 1 (Completely disagree)
   - 2 (Mostly agree)
   - 3 (Undecided)
   - 4 (Mostly disagree)
   - 5 (Completely agree)
10. Attending the training program helped me to communicate with people working with me (same structure) better:
    - 1 (Completely disagree)
    - 2 (Mostly agree)
    - 3 (Undecided)
    - 4 (Mostly disagree)
    - 5 (Completely agree)
11. Attending the training program helped me to communicate with people working around me (other structures) better:
    - 1 (Completely disagree)
    - 2 (Mostly agree)
    - 3 (Undecided)
    - 4 (Mostly disagree)
    - 5 (Completely agree)
12. Attending the training program helped me to collaborate with people working with me (same structure) more:
    - 1 (Completely disagree)
    - 2 (Mostly agree)
    - 3 (Undecided)
    - 4 (Mostly disagree)
    - 5 (Completely agree)
13. Attending the training program helped me to collaborate with people working around me (other structures) more:
    - 1 (Completely disagree)
    - 2 (Mostly agree)
    - 3 (Undecided)
    - 4 (Mostly disagree)
    - 5 (Completely agree)
14. Attending the training program helped me to improve relationships with superiors and top management:
    - 1 (Completely disagree)
    - 2 (Mostly agree)
    - 3 (Undecided)
    - 4 (Mostly disagree)
    - 5 (Completely agree)
15. Attending the training program helped me to collaborate with people working in other organizations more:
    - 1 (Completely disagree)
    - 2 (Mostly agree)
    - 3 (Undecided)
    - 4 (Mostly disagree)
    - 5 (Completely agree)
16. Attending the training program improved my career prospects:
    - 1 (Completely disagree)
    - 2 (Mostly agree)
    - 3 (Undecided)
    - 4 (Mostly disagree)
    - 5 (Completely agree)
17. Attending the training program will probably improve my career prospects:
    - 1 (Completely disagree)
    - 2 (Mostly agree)
    - 3 (Undecided)
    - 4 (Mostly disagree)
    - 5 (Completely agree)
